# 382. Vitamin D Supplementation and Covid 19: Results from the U.S. N3C Data Enclave

**DOI:** 10.1093/ofid/ofab466.583

**Published:** 2021-12-04

**Authors:** Kim Murray, Kathleen M Fairfield, Clifford james Rosen, Sally L Hodder, Jeremy Harper

**Affiliations:** 1 Maine Medical Center Research Institute, Portland, Maine; 2 Maine Medical Center, Portland, Maine; 3 West Virginia University School of Medicine, Morgantown, West Virginia; 4 Owl Health Works, Indianapolis, Indiana

## Abstract

**Background:**

It is estimated that 18% of adults in the U.S. take Vitamin D supplements. Some observational studies suggest that vitamin D supplementation activates the innate immune system and reduces the incidence and severity of viral infections. During the SARS-CoV-2 pandemic, vitamin D supplements were touted as a potential therapy to prevent the disease and/or complications. However, supportive evidence is lacking.

**Methods:**

The National COVID Cohort Collaborative (N3C) enclave is the largest COVID-19 data base with nearly 1.4 million positive patients at 56 sites in the U.S. We performed a retrospective analysis of vitamin D supplementation, either prescribed before or during hospitalization for SARS-CoV-2.

**Results:**

137,399 people took vitamin D supplements out of 1.4 million. Females prescribed vitamin D outnumbered males by almost 2:1, whereas in non-users there were no sex differences. Most supplement users were older than 50. African Americans constituted 13% of the non-users, but 23% of those prescribed vitamin D. Infected individuals with any vitamin D supplementation, pre-Covid, post-Covid or both, had a 6.66% mortality rate vs 2% mortality in non-users. Similarly, nearly a third of the supplement users were hospitalized compared to 11% in the non-users. The Charlson Co-Morbidity Index was 3.0±3 (SD) in users vs 1.0±2 (SD) in non-users.

**Conclusion:**

10% of SARS-CoV-2 infected patients were taking vitamin D. They tended to be older, more likely to be African American and have significant co-morbidities. Hospitalization and mortality were higher among those taking Vitamin D in this cohort. Vitamin D is widely used to prevent and treat SARS-CoV-2 but without evidence of efficacy.

**Disclosures:**

**Sally L. Hodder, M.D.**, **Gilead** (Advisor or Review Panel member)**Merck** (Grant/Research Support, Advisor or Review Panel member)**Viiv Healthcare** (Grant/Research Support, Advisor or Review Panel member)

